# Different Mechanisms Underlie the Metabolic Response of GBM Stem-Like Cells to Ionizing Radiation: Biological and MRS Studies on Effects of Photons and Carbon Ions

**DOI:** 10.3390/ijms21145167

**Published:** 2020-07-21

**Authors:** Alessandra Palma, Sveva Grande, Lucia Ricci-Vitiani, Anna Maria Luciani, Mariachiara Buccarelli, Mauro Biffoni, Valentina Dini, Giuseppe A. P. Cirrone, Mario Ciocca, Laura Guidoni, Roberto Pallini, Vincenza Viti, Antonella Rosi

**Affiliations:** 1 National Centre for Innovative Technologies in Public Health, Istituto Superiore di Sanità, 00161 Rome, Italy; alessandra.palma@iss.it (A.P.); sveva.grande@iss.it (S.G.); annamaria.luciani@iss.it (A.M.L.); valentina.dini@iss.it (V.D.); laguidoni@gmail.com (L.G.); vincefl.viti@gmail.com (V.V.); 2 Department of Oncology and Molecular Medicine, Istituto Superiore di Sanità, 00161 Rome, Italy; lucia.riccivitiani@iss.it (L.R.-V.); mariachiara.buccarelli@iss.it (M.B.); mauro.biffoni@iss.it (M.B.); 3 Istituto Nazionale di Fisica Nucleare INFN Sez. di Roma, 00185 Rome, Italy; 4 National Institute for Nuclear Physics, Laboratori Nazionali del Sud, INFN-LNS, 95123 Catania, Italy; pablo.cirrone@lns.infn.it; 5 Centro Nazionale di Adroterapia Oncologica (CNAO)-National Center for Oncological Hadrontherapy, 27100 Pavia, Italy; mario.ciocca@cnao.it; 6 Department of Neuroscience, Fondazione Policlinico Universitario A. Gemelli, Università Cattolica del Sacro Cuore, 00168 Rome, Italy; roberto.pallini@unicatt.it

**Keywords:** glioblastoma, stem cells, metabolism, MRS, photon beams, carbon ions

## Abstract

Glioblastoma multiforme (GBM) is a malignant primary brain tumor with very poor prognosis, high recurrence rate, and failure of chemo-radiotherapy, mainly due to a small fraction of cells with stem-like properties (GSCs). To study the mechanisms of GSCs resistance to radiation, two GSC lines, named line #1 and line #83, with different metabolic patterns and clinical outcome, were irradiated with photon beams and carbon ions and assessed by ^1^H Magnetic Resonance Spectroscopy (MRS). Both irradiation modalities induced early cytotoxic effects in line #1 with small effects on cell cycle, whereas a proliferative G2/M cytostatic block was observed in line #83. MR spectroscopy signals from mobile lipids (ML) increased in spectra of line #1 after photon and C-ion irradiation with effects on lipid unsaturation level, whereas no effects were detected in line #83 spectra. Gamma-Aminobutyric Acid (GABA), glutamic acid (glu) and Phosphocreatine (pCr) signals showed a significant variation only for line #1 after carbon ion irradiation. Glucose (glc) level and lactate (Lac) extrusion behaved differently in the two lines. Our findings suggest that the differences in irradiation response of GSCs #1 and #83 lines are likely attributable to their different metabolic fingerprint rather than to the different radiation types.

## 1. Introduction

Glioblastoma multiforme (GBM) is the most common malignant glioma in adults. Despite innovative research efforts for targeted therapies, the outcome remains poor [1]. To enhance sensitivity to radiotherapy (RT), the mechanisms underlying the different cell response to radiation need to be further elucidated and new strategies developed [2,3].

The cancer stem cell (CSC) hypothesis attributes treatment failure to a small fraction of self-renewing cells with stem-like properties and high resistance to radiation [4,5] that seems to be responsible for tumor recurrence in GBM. Because of the high resistance to photon beam irradiation [6], several studies have examined the response of CSCs to irradiation with proton beams and charged particles. In particular, heavy ion radiotherapy has been shown to have potential advantages over photons in treating many radioresistant human colon cancers [7]; a recent paper shows that the combination of proton therapy and a radiosensitive compound could overcome GBM resistance to conventional treatments [8]. However, results from clinical trials conducted through a comparative analysis between protons, carbon ions and photon beam (Cinderella and Cleopatra), are still pending [9,10,11]. A Phase II clinical trial on selected GBM patients used a boost of C-ion RT prior to the initiation of the standard treatment course [12]. Using a 3D model for investigating GSC radiosensitivity to proton beam and carbon ion irradiation, Chiblack showed an enhanced biological effectiveness of C-ion RT in vivo, due to its potent antiangiogenic effects and eradication of radioresistant hypoxic tumor cells [13,14]. As other cancers, GBM displays high heterogeneity among patients, with relevant differences in genomic, transcriptomic, proteomic, and metabolomic features, and quite different cell populations seem to be present in the same tumor [15,16]. Both inter- and intra-individual heterogeneity may then be responsible for treatment failure and tumor relapse, suggesting the necessity of tailored therapies. In previous studies, subtypes of high-grade glioma have been identified on the basis of molecular gene expression [17] which includes the proneural, proliferative, and mesenchymal subtypes. The hypothesis that the ability to escape or to mitigate radiation damage could be due to different subtype characteristics deserves to be investigated. Further profiling studies revealed the presence of two distinct subsets of GSCs based on gene expression analysis: (a) a subset, displaying a Glioblastoma full stem-like phenotype (GSf-like), highly tumorigenic and invasive in vivo, and an enriched proneural gene expression signature; (b) the other with a Glioblastoma restricted stem-like phenotype (GSr-like) showing expression signatures more similar to stabilized cell lines than to the original patient tumors [18,19]. Moreover, it has been shown that stratification of GSCs according to their metabolic MRS signals parallels the genetic profiles typical of GSf/GSr cell lines [18]. Finally, GSCs were characterized by high heterogeneity [20] since cells with the same brain origin and similar clinical parameters belonged to different metabolic clusters and showed different genetic signatures [18,19,21]. These results led to investigate if the response to irradiation in different GSC lines could be due to different genetic and metabolic fingerprints rather than to the radiation type. 

Metabolomics and metabolic profiling, as biochemical picture of a particular phenotype, can be explored by MRS as a tool to further understand metabolic changes occurring during tumor initiation, progression, and radiotherapy resistance [15]. Improved knowledge of the metabolism of brain tumors may contribute to discover new diagnostic or prognostic biomarkers, overcoming the problem of poor prognosis. In particular, metabolic reprogramming is a hallmark of GSCs [22] used to identify malignance signatures and open new ways to develop tailored therapeutic approaches. Stratifying patients according to molecular biomarker profiles is a key step to manage patient heterogeneity.

To study the mechanisms involved in GSCs radiation resistance and evaluate whether their response to irradiation is due to different radiation type or to intrinsic GSCs characteristics, two GSC lines, named #1 and #83, were selected on the basis of their metabolic profiles and genetic signatures, deeply analyzed in previous studies [18,21]. Particularly a mixed neural–astrocyte metabolic phenotype with a strong neuronal fingerprint was found in line #1, while a glioma-like metabolism prevailed in line #83. We performed MRS experiments on these cells after irradiation with ^137^Cs photons and C-ions (CNAO, Pavia, Italy and INFN-LNS, Catania, Italy) at different Linear Energy Transfer (LET) value at a dose of 20 Gy. Effects on cell survival and cell cycle were also evaluated.

## 2. Results

**Figure 1:**
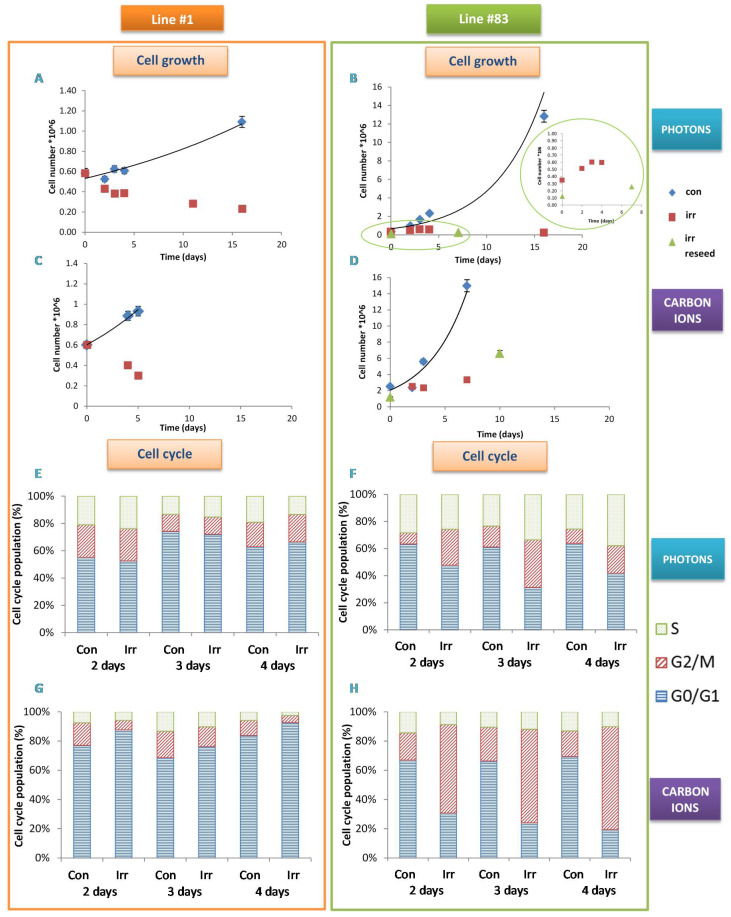
Irradiation affects growth and cell cycle of line #1 and #83 in a different way. Growth curves of line #1 (**A**,**C**) and of line #83 (**B**,**D**) after photon and carbon ion irradiation at 20 Gy. For line #83 values of cell growth after reseeding (“irr reseed”) are also reported. In panel B the detailed expanded region of cell growth for line #83 is shown. Exemplificative experiments are reported; error bars correspond to measurement error (5%). Distribution of cell cycle phases for line #1 (**E**,**G**) and for line #83 (**F**,**H**) after photon and carbon ion irradiation at 20 Gy as a function of time after irradiation (Con: control and Irr: irradiated sample).

GSC lines #1 and #83 were selected from a panel of GSCs on the basis of their biological and metabolic characteristics [20,21]. Although they both derived from tumors localized close to the temporal subventricular zone, they have different overall survival (12.5 and 8.5 months for line #1 and line #83, respectively), different CD133 positivity (more than 80% in line #1 and less than 1% in line #83); moreover line #1 cells grow as neurospheres, while line #83 cells grow mostly as monolayer. Finally, they belong to different metabolic and genetic clusters [18,19,20,21]. Cell growth of both control and 20 Gy photon irradiated lines are shown in Figure 1A,B. A cytotoxic effect was observed for line #1 at short and late times (A), while only a cytostatic effect was observed in early times of growth for line #83 cells (B). These latter, in late times, maintain their growth in culture, and resume proliferation once they have been re-seeded, (see Inset in Figure 1B). A similar behavior was observed after irradiation with carbon ions (Figure 1C,D). Cell growth data were confirmed by the trend of the cell cycle in of the two lines. The line #1 cells after carbon ion irradiation accumulated in G0/G1 phase due to a proliferation arrest (Figure 1G). Moreover, the percentage of cells in S phase decreased in all carbon ion irradiated samples and at late time after photon irradiation (Figure 1E,G). Differently, cells from line #83 were significantly blocked after both photon and carbon ion irradiation in G2 phase where cell damage repair is known to occur (Figure 1F,H).

Apoptosis for both lines is shown in Supplementary Figure S1. Apoptosis onset was observed in line #1 by 2 days after cell irradiation, then apoptosis increased at 3 days, becoming relevant at 4 days after irradiation. For cell line #1 mainly necrosis gave a relevant contribution to cell death. On the contrary in line #83 the percentage of dead cells was smaller in the time range studied (Figure S1).

Metabolomics and metabolic profiling of the two cell lines have been explored by MRS. As already reported, the analysis of metabolic profiles for lines #1 and #83 indicated that these two lines strongly differ in several metabolites as shown in box plots and Fold Change evaluations shown in Figure S2. 

**Figure 2:**
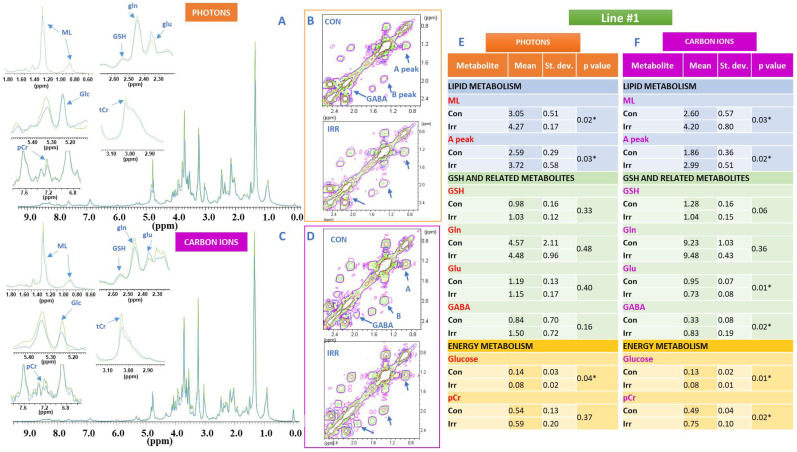
^1^H NMR spectra of line #1 cells, signals of interest and metabolic modifications induced by radiation treatment. (**A**,**B**) 1D and 2D spectra of photon beam irradiated line #1 cell samples (green trace) and corresponding controls (blue trace) together with the spectral regions of interest for the analysis: Mobile Lipids (ML, 1.28 ppm), glutathione and related metabolites (GSH 2.55 ppm, gln 2.45 ppm, glu 2.35 ppm), glucose (glc 5.22 ppm), total creatine (tCr, 3.02 ppm) and phosphocreatine (pCr, 7.25 ppm). In panel B cross peak A, arising from the correlation of CH_3_-terminal methyl group of Fatty Acid (FA) chains with the bulk of FA (excluding omega-3 FAs), at (0.89–1.28) ppm, cross peak B, representing both mono and polyunsaturated FA at (2.02–1.28) ppm and GABA cross peak at (2.30–1.90) ppm are labelled. (**C**,**D**) 1D and 2D spectra of carbon ion irradiated samples (green trace) at 20 Gy and corresponding controls (blue trace) of lines #1 and #83. Region of interest were expanded. All spectra were acquired 48 h after irradiation at a dose of 20 Gy with both photons and carbon ions. Assignments were performed as reported in [21]. Tables (**E**) (photon beams) and (**F**) (carbon ions) show mean values of all analyzed metabolites signal intensities, standard deviations and *p* values, calculated on at least 5 experiments performed 48 h after irradiation and corresponding controls. Stars indicate statistically significant variations (*p* value < 0.05).

**Figure 3:**
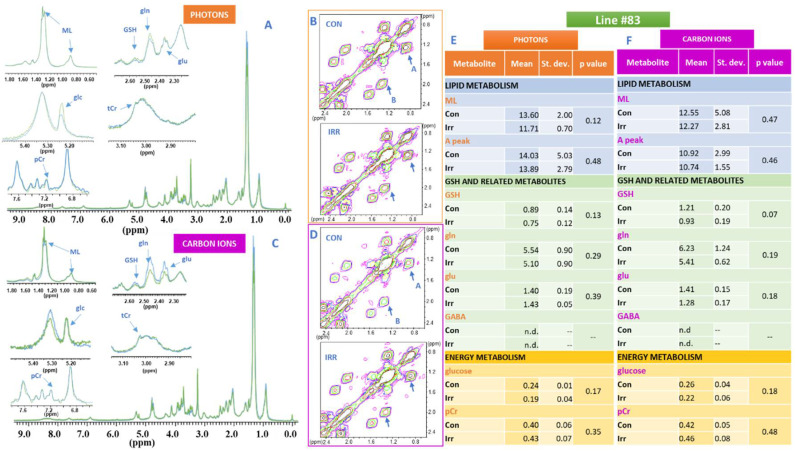
^1^H NMR spectra of line #83 cells, signals of interest and metabolic modifications induced by radiation (**A**,**B**) 1D and 2D spectra of photon beam irradiated line #83 cell samples (green trace) and corresponding controls (blue trace) together with the spectral regions of interest for the analysis: Mobile Lipids (ML, 1.28 ppm), glutathione and related metabolites (GSH 2.55 ppm, glu 2.35 ppm, gln 2.45 ppm), glucose (glc 5.22 ppm), total creatine (tCr, 3.02 ppm) and phosphocreatine (pCr, 7.25 ppm). In panel B the cross peak A, arising from the correlation of CH_3_-terminal methyl group of Fatty Acid (FA) chains with the bulk of FA (excluding omega-3 FAs), at (0.89–1.28) ppm, and cross peak B, representing both mono and polyunsaturated FA at (2.02–1.28) are labelled. Cross peak diagnostic for GABA is barely detectable (not shown). (**C**,**D**) 1D and 2D spectra of carbon ions irradiated samples (green trace) at 20 Gy and corresponding controls (blue trace) of lines #1 and #83. Region of interest were expanded. All spectra were acquired 48 h after irradiation at a dose of 20 Gy with both photons and carbon ions. Assignments were performed as reported in [21]. Tables (**E**) (photon beams) and (**F**) (carbon ions) show mean values of all analyzed metabolites signal intensities, standard deviations and *p* values, calculated on at least 5 experiments performed 48 h after irradiation and corresponding controls.

The whole MR spectra of the two lines, the analysed metabolic signals (lipid, glutathione and energy metabolism), selected region of 2D COSY spectra and the corresponding intensity values are shown in Figure 2 and Figure 3 for line #1 and line #83, respectively. The MRS spectral signals were assigned according to [21].

In particular, 1D and 2D spectra of photon beam irradiated samples (green trace) and their controls (blue trace) of lines #1 and #83 together with the spectral regions of interest are shown in A and B panels of Figure 2 and Figure 3, respectively, where changes induced by irradiation on the analysed metabolites can be observed. Similarly, 1D and 2D spectra of carbon ion irradiated samples (green trace) and their controls (blue trace) of lines #1 and #83 and the regions of interest where metabolite changes can be detected are shown in C and D panels of Figure 2 and Figure 3, respectively. Spectra were acquired 48 h after irradiation.

Mean values of lipid, glutathione and energy metabolites signal intensities, standard deviations and *p* values, calculated on at least 5 samples, are shown in Figure 2E (photon beams) and Figure 2F (carbon ions) for line #1 and Figure 3E (photon beams) and Figure 3F (carbon ions) for line #83, respectively. 

The intensities of signals from lipids (ML) increased in spectra of line #1 after both photon and C ion irradiation (Figure 2A,C,E,F), while remain unaffected in spectra from line #83 (Figure 3A,C,E,F). The behavior of lipid signals was also monitored as cross-peak A in 2D spectra from lipid chains giving the same results for both lines (Figure 2B,D–F; Figure 3B,D–F). It is worth noting the intrinsic high level of ML in line #83 as reported in Figure S2A,B. Clone analysis for line #83 is shown in Figure S2D to testify the higher heterogeneity of this line with respect to line #1. 

**Figure 4:**
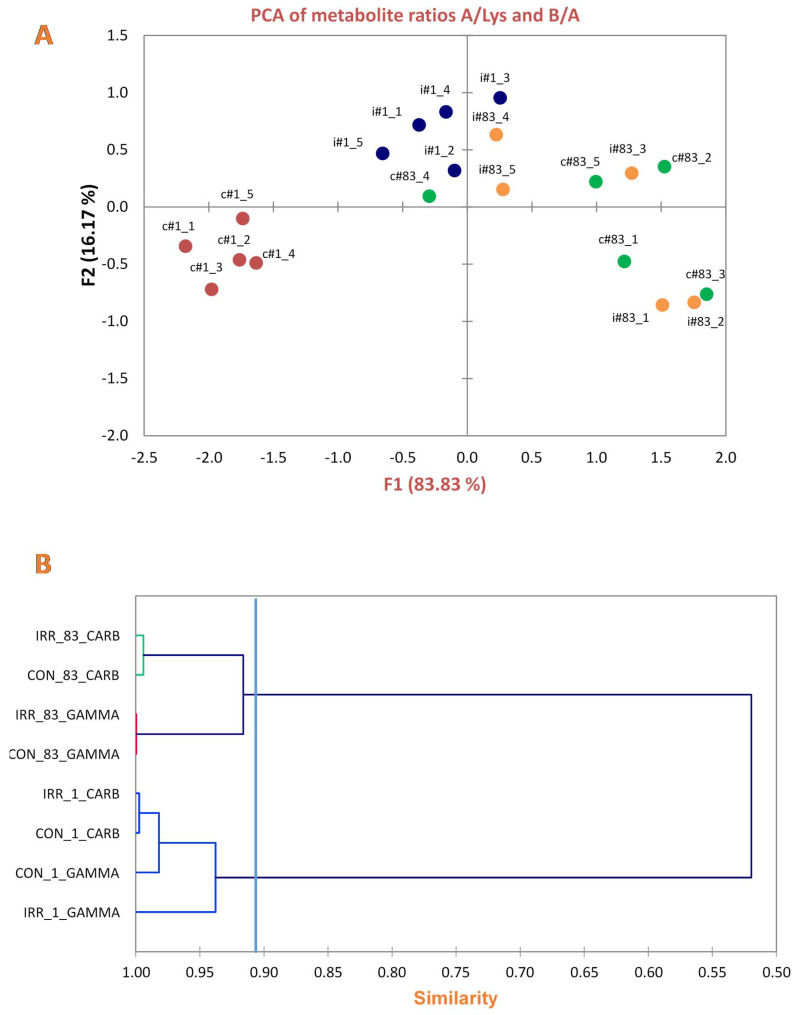
Statistical analysis confirms a different metabolic behaviour of line #1 and #83 cells after irradiation (**A**) F1 vs. F2 plot from Principal Component Analysis of observations of ML cross peak A and cross peak ratio B/A from 2D NMR spectra, for line #1 and #83. Peak A at (0.89–1.28) ppm arises from the correlation of CH3-terminal methyl group of FA chains with the bulk of FA (excluding omega-3 FAs). Peak B at (2.02–1.28) ppm represents both mono and polyunsaturated FA with a different contribution from n-9 and n-3 chains. F1 and F2 are the first and the second components in the PCA space, that account for 83.83% and 16.17%, respectively representing 100% of the total variances existing along the obtained data. Each point in the plot corresponds to the mean value of at least three experiments at different times after irradiation. (**B**) Dendrogram resulting from Unsupervised Cluster Analysis of GSH, gln, glu and GABA mean signal intensities from experiments at different times after irradiation.

It is interesting to note that the level of lipid unsaturation, measured as the B/A ratio of cross peak intensities (Figure 2B and Figure 3B), was strongly influenced in line #1 with respect to line #83 (Figure 4A). Changes in the B/A ratio are in fact indicative of the increasing level of unsaturated fatty acids compared to total FA. Principal component analysis (PCA) shows that the control values of line #1 are always separated from those irradiated, independently of the radiation type, while in line #83 there is no statistically significant difference between control and irradiated samples (Figure 4A).

The signals of GSH, gln, glu and GABA were influenced in line #1 and line #83 after irradiation with photons and carbon ions, though to a different extent (Figure 2 and Figure 3, respectively). In particular, in line #1 the intensity of the glu signal decreases, while the GABA signal increases in intensity in a statistically significant way only for carbon ion irradiation, whereas no statistically significant effects were observed for these signals in line #83, where the GABA signal was not detectable (Figure 2 and Figure 3).

Unsupervised Cluster analysis of GSH, gln, glu signal intensities from spectra was also performed. The resulting dendrogram is shown in Figure 4B. As expected, the two lines were grouped in different cluster cutting the tree at appropriate level. Control and irradiated samples of lines #1 and #83 are grouped together independently of the type of irradiation.

In addition, the signals of phosphocreatine (pCr) and total creatine (tCr) in line #1 spectra were strongly influenced by irradiation with carbon ions (Figure 2C,F), while no statistically significant effects were observed in the spectra of line #83 (Figure 3A,C,E,F).

**Figure 5:**
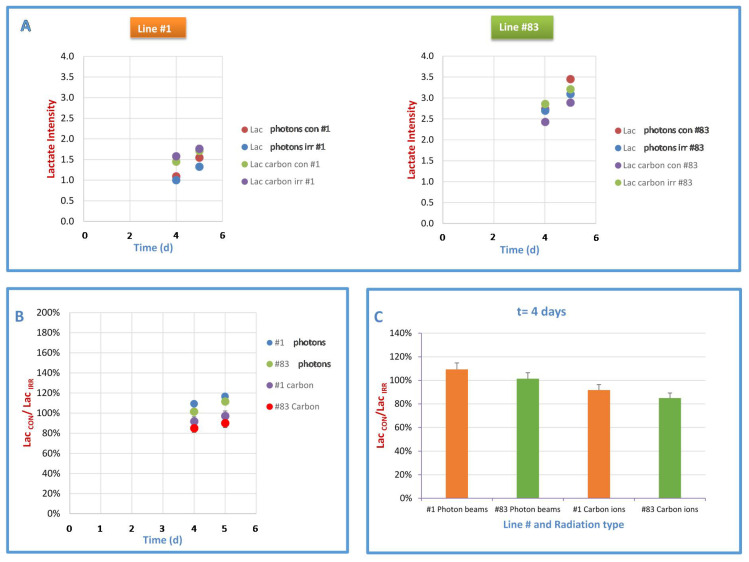
Lactate signal and cell number after photon and carbon ion irradiation. (**A**) lactate extrusion in culture medium, after photon and carbon ion irradiation at 20 Gy, as a function of time, for control and irradiated samples of line #1 and of line #83. Lactate doublet signal at 1.44 ppm was assigned and valuated in growth media spectra according to (Grande 2016) (**B**) Lac_con_/Lac_irr_ ratio from lines #1 and #83 culture media spectra after irradiation with photons and carbon ions at 20 Gy as a function of time. (**C**) Lac_con_/Lac_irr_ values calculated at 4 days after irradiation, indicative of the entity of Warburg effect for both lines.

Finally, the intensity of the glucose signal (glc) clearly decreased in the spectra of line #1 after irradiation with photon and carbon ions (Figure 2A,C,E,F), while the decrease of glc signal intensity in line #83 spectra after irradiation was not statistically significant (Figure 3A,C,E,F). The lactate signal from spectra of culture media of the two cell lines increased in time, but only minor changes were observed after photon and carbon ion irradiation (Figure 5A). Lac_con_/Lac_irr_ ratio for both types of irradiation in both lines increased as a function of time, (Figure 5B), being always greater than 100% for photons and less than 100% for carbon ions. This ratio, that can be used as an indication of the Warburg effect entity according to what reported in [23], is significantly higher in line #1 than in line #83, especially taking into account the lower cell number of line #1 with respect to line #83. This indicated an increased anaerobic glycolysis as confirmed by increased Warburg effect (Figure 5B,C, where a snapshot of the effect at 4 days after irradiation is shown).

Effects on the selected metabolites after irradiation with both photon beams and carbon ions as a function of time are reported in Figure S3 only for line #1, where statistically significant differences were observed. These effects remained stable until 4 days after irradiation (Figure S3).

Finally, a synthesis of the metabolic response of the two GSC cell lines to photon and C-ion irradiation is reported in Table 1 together with their genetic and metabolic different profiles. Line #83 metabolism is not affected by irradiation with photons and Carbon ions. These cells undergo a G2 block more intense after Carbon ion irradiation, but the ability to recover is similar in both cases. Differently line #1 seems to be more responsive to Carbon ions with respect to photons, but in both cases, cells die and are not able to recover in any way.

## 3. Discussion

In GBM the self-renewing, tumorigenic GSC population likely contributes to tumor initiation and therapeutic resistance. In addition, as normal stem and progenitor cells participate in tissue development and repair, these developmental strategies re-emerge in GSCs to support the progressive growth of tumors [5,17,24,25,26,27,28,29,30,31,32,33]. Several strategies have been proposed to investigate the mechanisms leading to GSC escape from therapeutic treatments. Charged ion therapy was proposed [13,14,34,35] to overcome GSC radiation resistance because of its potential greater effectiveness than photon therapy, but no definitive results have been obtained. Emerging evidence shows that metabolic reprogramming of glioblastoma mediates resistance to standard therapies [36]. Glioblastoma is associated with a significant increase in glycolysis and knockdown of glycolytic genes strongly inhibits GBM growth [37]. The high utilization of glucose suggests that inhibiting glycolysis may have a therapeutic value. Also, isocitrate dehydrogenases (IDHs) metabolism plays a crucial role in the tricarboxylic acid cycle; in IDH wild type (wt) GBM, the upregulation of wtIDH1 (wild type isocitrate dehydrogenase 1) fuels therapy resistance [36]. It is known that glutamine levels are higher in GBM tissue than in the surrounding normal brain and that the ability to metabolize glutamine is critical for GBM proliferation and survival. Elevated glutaminase and glutamate levels following mTOR (mammalian target of rapamycin) kinase inhibitor treatment promoted GBM survival while combined inhibition of mTOR kinase and glutaminase resulted in massive synergistic tumor cell death and growth inhibition. These results highlight a critical role for compensatory glutamine metabolism [38].

High GBM heterogeneity seems to play a relevant role in GSC ability to escape therapies. A recent study that used single-cell RNA sequencing of primary GBMs revealed extraordinary diversity within each tumor [39]. The high level of heterogeneity in GBM has been recently invoked [39,40] as one of the reasons for treatment failure and tumor relapse. Stratifying patients according to molecular biomarker profiles is a key step to manage patient heterogeneity.

Metabolomics may help in clarifying stem cell fate and reprogramming function [41,42]. In the present study two GSC lines, #83 and #1, characterized by different metabolic profiles [21] and clone heterogeneity [20] were analyzed as response to photons and carbon ions in terms of effects on cell survival, cell cycle and metabolism. Highly heterogeneous clones of line #83 [20] may be responsible for different responses to irradiation if compared to homogeneous clones of line #1. Indeed, while #83 clones 1 and 2 resulted similar to the origin line #83, significant differences in clone 3 were observed for NAA and Myo metabolites and in clones 4 and 5 for lipid concentration. Finally, in clone 5 the alfa amino-adipic-acid signal, previously correlated to poor prognosis [43], was observed.

A previous study on 52 GSC lines analyzed in terms of LD50 after photon irradiation reported a similar response to a 20 Gy irradiation for both lines [6]. However, in the present study the two cell lines behave differently after irradiation with both photons and carbon ions; only line #83 cells are able to recover after carbon ions and photon beam irradiation, in agreement with preliminary data on cell survival. The presence of a plateau in the survival levels after charged particles and photon irradiation at doses higher than 10 Gy, suggested the presence of radio-resistant subpopulations in this GSC line [44]. According to this hypothesis, some of the heterogeneous clones from line #83 [20] may be able to recover and to reestablish the original pattern, differently from line #1 cells that do not show such heterogeneity. The different response to radiation treatments of the two lines here examined in terms of cell cycle and metabolic changes may be attributable mainly to the intrinsic characteristics of the lines, rather than to radiation type.

Cell cycle checkpoints and mechanisms involved in DNA repair are clearly interdependent, with the choice of repair mechanism consistently adjusted throughout the cell cycle [45,46]. Existing models established in different cell types have shown that radio-induced double-strand breaks (DSB) undergo faster repair in the G2 phase during the first hours following irradiation [47]. Additionally, particle therapy is effective at cell killing irrespective of the cell cycle phase, unlike photons [48,49], where cells are more resistant in late S and G2 phase [46,50]. It is still unclear if cells preferentially select specific pathway to repair DSBs generated by high LET radiation, unlike low LET radiation-induced DSBs. The arrest of the line #83 cells in the G2/M phase, where DNA repair processes are known to occur, may explain the higher radioresistance of this cell line, differently from cell line #1, that undergo cytotoxic effect without relevant G2/M arrest. The effect on line #83 cells seems more relevant after carbon ion compared to photon irradiation. This may be due to higher Relative Biological Effectiveness (RBE) value able to induce less reparable damages in agreement with the role played by DSBs in high LET particle irradiation. In this situation a higher permanence of cells in G2 phase may allow them to better repair and to recover at late times after irradiation.

Lipids detected as Mobile Lipids play a relevant role in GSC escaping mechanisms to radiotherapy. The lipid signal increased in line #1 after irradiation, while only small fluctuations could be detected in line #83. This increase in MLs in line #1 can be attributed to the accumulation of lipids in the cytoplasm as a consequence of metabolism impairment [51,52]. In contrast, in line #83, MLs are segregated and stored mainly in cytoplasmic lipid droplets where they are protected from oxidation and from the formation of unstable lipid peroxides, which are toxic to cells [53]. It has been recently reported [54] that lipolytic inhibitor G0/G1 switch gene 2 (G0S2) is upregulated in radioresistant GSCs and elevated in clinical GBM. GBM patients with high G0S2 expression had significantly shorter overall survival compared with those with low expression of G0S2. Moreover, G0S2-induced radioresistance is related with G0S2-mediated lipid droplet stability. The intrinsic notable difference in lipid content (high in line #83, low in line #1) of the two lines is consistent with this hypothesis and with the different patient survival.

The two lines belong to distinct metabolic clusters, where lipid content is one of the important metabolic parameters differentiating mesenchymal from neuronal fingerprint [20,21]. The different effects after photon or carbon ion irradiation in the two lines support the hypothesis of a metabolic switch of line #1 cells towards the mesenchymal fingerprint and not a mechanism to protect themselves from irradiation. This interpretation is supported by the observation of the increase in unsaturation of lipid chain in irradiated line #1 towards more typical value of line #83. Our results highlight the physiological relevance of Lipid Droplets in lipid metabolic reprogramming of cancer cells [55].

As GSH is concerned it is well known the role of this metabolite as the most important active oligopeptide in the maintenance of redox homeostasis, with a radiation protection role against oxidative stress. The absence, after irradiation, of relevant changes of this metabolite in both cell lines shows that GSH is not a key metabolite in protecting GSCs from irradiation. On the contrary, the GABA increase, concomitantly with a glu decrease in irradiated cells from line #1, is particularly relevant after carbon ion irradiation and suggests a GABA synthesis increase. Indeed, GABA is synthesized via enzymatic decarboxylation of l-glutamate involving glutamic acid decarboxylase (GAD) and pyridoxal-l-phosphate (PLP) as cofactor [56]. Unfortunately, changes of oxygenation do not clearly differentiate conditions for GABA increase/decrease, as its increase was observed after continuous hypoxia in neurons, but not in glia while intermittent hypoxia would inhibit the GAD enzyme activity [56]. Noteworthy, a powerful eradication of radioresistant hypoxic tumor cells was attributed to carbon ion irradiation [57]. The GABA increase in line #1 cells, evident only after carbon irradiation, could be related to such effect, confirming a non-complete eradication of radioresistant hypoxic cells also due to the absence of the protecting agent against irradiation GSH. 

Cancer cells have significant heterogeneity in glucose metabolism. Dysfunction of mitochondria, loss of tumor suppressors, the hypoxic microenvironment, and oncogene-driven metabolic reprogramming are initiating events of abnormal energy metabolism in cancer cells [58]. At present a widely accepted hypotheses suggests that energy metabolism is strictly involved taking into account that one of the established features of cancer is the deregulation of cellular energetics [59]. Glycolysis represents an important tool for tumor cells driving the malignant progression [60]. Particularly GBM, like most malignant solid tumors, is characterized by high levels of glycolysis with large amounts of lactic acid production and many reports have identified the Warburg effect to be implicated in resistance to cytotoxic stress induced by radiotherapy. In line with this hypothesis, in a previous study [20] it has been shown that in line #83, where aerobic glycolysis is predominant, the glucose signal remains unchanged after irradiation. On the contrary, glucose decrease after both types of irradiation for line #1 is likely due to a shift from OXPHOS to aerobic glycolysis, suggested as typical cell response to external challenge [58] and the consequent rise of the Warburg effect in this line, confirmed by the higher decrease of the ratio Lac_con_/Lac_irr_.

In tumor cells, glycolysis is the main supplier of intracellular ATP and cytosolic phosphocreatine-creatine kinase (CK) was found associated with enzymes of glycolysis [61,62,63] though the role of CK in glycolysis has not been fully elucidated. The CK shuttle system is a key metabolite in the brain and central nervous system networks. In line #1 pCr signal increased by irradiation with carbon ions for a possible impairment of the CK shuttle system, likely impairing cytosolic CK by oxidative and radical damage [64]. As pCr is involved in energy exchange, it is possible to hypothesize a contribution of the pCr metabolic pathway in line #1, differently to what observed for line #83. All these effects on energetic metabolism could be hypothesized as a further mechanism contributing to the higher radiosensitivity of line #1 compared to line #83.

In conclusion metabolism of line #83, analyzed by MRS, is unresponsive to irradiation, while in line #1 cells alterations of metabolic patterns of FA, GABA, pCr, and glc confirm the involvement of cell metabolism, including energy pathways, in cell radiosensitivity. Differences in radiation response may, therefore, be likely attributed to genetic and metabolic differences between the two lines, representative of mesenchymal and proneural profiles, regardless of radiation type. Further experiments are foreseen to have more insight in the observed effects. In this respect, proton MRS may contribute to study cell metabolism in GSCs with different metabolic and genetic signatures after different radiotherapeutic approaches.

## 4. Materials and Methods

### 4.1. Patients and Tumor Characterization

Tumor tissue samples were collected from adult patients with GBM tumors (WHO grade IV) undergoing complete or partial surgical resection at the Institute of Neurosurgery, Catholic University School of Medicine in Rome. Informed consent was obtained from the patients before surgery. All patients provided written informed consent according to the research proposals approved by the Ethical Committee of the Catholic University School of Medicine, UCSC (Prot. 4720/17). Patients were eligible for the study if a diagnosis of glioblastoma multiforme was established histologically according to the WHO classification [65]. Details about patient’s treatments and GSCs isolation were given elsewhere [21,66]. Experimental protocols were approved by the ethical committee of the Catholic University of Rome.

The expression of the proliferation marker Ki-67 and of Phosphatase and Tensin Homolog were characterized on tumor specimen by immunohistochemistry on deparaffinized sections using the avidin-biotin-peroxidase complex methods (ABC-Elite Kit, Vector Laboratories, Inc., Burlingame, CA, USA), anti-Ki67 monoclonal antibody (MIB-1, Dako, Agilent, Santa Clara, CA, USA) and anti-Phosphatase and Tensin Homolog mouse monoclonal antibody (clone 28H6; Novo Castra, Newcastle, UK). O6-methylguanine-DNA methyltransferase promoter methylation patterns were assessed on genomic DNA extracted from paraffin-embedded tissue by methylation-specific PCR as previously described [66,67]. Levels of VEGF and EGFRvIII were assessed as previously described [66]. 

### 4.2. Glioblastoma Stem-Like Cell Cultures

GSCs were isolated through mechanical dissociation of the tumor tissue and cultured in a serum-free medium supplemented with epidermal growth factor and basic fibroblast growth factor as previously described [66]. The in vivo tumorigenic potential of GBM neurospheres was assessed by intracranial or subcutaneous cell injection in immunocompromised mice. GSC lines were validated by Short Tandem repeat DNA fingerprinting as previously described [68,69].

Cell number in biological experiments has been evaluated by a Scepter 2.0 Coulter Counter (Merck, Darmstad, Germany).

Clones from GSCs were obtained by plating single cells into 96 wells plate. After 4 weeks, single clones were mechanically dissociated and replated to expand the culture.

For irradiation experiments GSCs were seeded to be expanded. At 3th passage cells were pooled and stored as 1 × 10^6^ cells/vial in nitrogen and 50 vials were prepared to be used for all the experiments at identical starting conditions.

Seven days before the experiments, the GSC #1 and GSC #83 cell lines were seeded in T175 cm^2^ flasks at a density of about 4 × 10^4^/mL and 2 × 10^4^/mL in 50 mL total volume, respectively. Just before irradiation, cells were collected and placed in different sterilized sample holders, depending on the irradiation facility used: commercial 1 mL polystyrene cuvettes (INFN-LNS and ISS) and commercial UVette (CNAO). These sample holders were then centrifuged at low speed to obtain cell pellets 4 mm and 2 mm thick, respectively.

### 4.3. Irradiation Conditions

Irradiation with C-ions was carried out at the multidisciplinary irradiation beamline facility of the INFN-LNS in Catania and at the CNAO hadrontherapy facility in Pavia. 

At the INFN-LNS, 62 AMeV in energy carbon ions, exited in air and reached the entrance of the cell suspensions at 52 MeV/u, corresponding to a Linear Energy Transfer (LET) value of 42 keV/µm. However, the thickness of the 4-mm cell pellet does affect the LET of C-ions, with a significant variation in the average LET from 42 to 56 keV/µm. Absolute dosimetry was performed following the IAEA TRS 398 Code of Practice using a plane parallel PTW Markus ionisation chamber [70]. At the CNAO facility, a 22 mm homogeneous Spread-Out-Bragg-Peak (SOBP) was obtained by C-ion beams of initial energies ranging from 187 to 214 MeV/u. Samples were placed in sealed vessels at the depth of 82 mm in water, in order to achieve an average LET value of 83 keV/µm, with no significant variation between the entrance and the exit of the 2 mm cell pellet (LET of 80 and 86 keV/µm, respectively).

^137^Cs γ-rays were obtained from a Gammacell 40 Exactor (Nordion) at the ISS, Rome, with a dose rate of about 0.8 Gy min^−1^.

All irradiations were performed at room temperature, with a single high radiation dose of 20 Gy comparable with: total doses delivered during radiation therapy in a fractionated regimen, single high doses used in other therapeutic modalities (i.e., intraoperative radiotherapy and stereotactic radiosurgery), and the boost before the chemo-radiotherapic treatments used for the treatment of patient with Carbon ions [12]. The same experimental protocols were used for charged particles and photon irradiations. Cell suspensions were centrifuged in 1-mL polystyrene cuvettes to obtain a 4-mm thick pellet, and irradiated; after irradiation, samples were prepared as in [20] for biological end points and MRS experiments.

### 4.4. ^1^H MRS Cell Sample Preparation

Cells were removed, washed in Phosphate-buffered saline (PBS) and centrifuged at 162 rcf for 3 min. The pellet was suspended in PBS with 20% D_2_O and 2 mM Sodium 3-(trimethylsilyl) propionate-2,2,3,3-d4 (TMSP) used as a frequency standard. A 15 μL aliquot of the suspension was inserted into a 1 mm MRS microtube and centrifuged to obtain a packed cell volume. 

Conditioned growth media, collected from cell cultures, were added with 20% D_2_O and 2 mM TMSP and transferred into a 1 mm MRS microtube. All MRS reagents were purchased from Cambridge Isotope Laboratories, Inc., Tewksbury, MA, USA.

### 4.5. ^1^H MRS Measurements

^1^H MRS experiments were run on a digital Bruker Avance spectrometer (Bruker Scientific LLC, Billerica, MA, USA) at 400.14 MHz, equipped with a 1mm microprobe. Both one dimensional (1D) and two-dimensional Correlation SpectroscopY (2D COSY) experiments were performed, at T = 298 K.

1D ^1^H MRS spectra of GSCs and culture media were acquired with a 90° RF pulse, the number of scans (ns) was equal to 1000 (sufficient to obtain a good signal-to-noise ratio) for cell spectra while ns = 4000 was used for culture media spectra. When indicated, a Lorentzian-Gaussian function was applied in the time domain, before Fourier transformation.

2D COSY spectra were acquired with a 90°-t1–90°-t2 pulse sequence and ns = 32 for cell or ns = 128 for culture media samples. Spectra were acquired as a matrix of 512 × 128 data points in time domain.

NMR parameters were obtained in at least three independent experiments and data are expressed as mean ± standard deviation (SD) values. WINNMR software (Bruker Scientific LLC, Billerica, MA, USA) was used to perform 1D signal deconvolution and 2D cross peak integration as reported in [21]. Macromolecule signal at 0.89 ppm intensity was used as internal reference for 1D measurement, while 2D signal integrals were normalized to the intensity of the lysine (Lys) cross-peak at 1.70–3.00 ppm. This peak was considered representative of the cellular mass, as it was found to be constant in a number of cell models and tissue samples [21].

### 4.6. Statistical Analysis

Unsupervised agglomerative hierarchical Clustering, Principal Component Analysis and Student’s t test were performed utilizing XLSTAT software (Addinsoft^TM^ Paris, France) version 2012.2.02.

## Figures and Tables

**Table 1:** GSC lines metabolic and biological response to photon and carbon ion irradiation at 20 Gy.

	GSC#1		GSC#83	
Genetic Profile [18]	GSf-Like		GSr-Like	
Metabolic Cluster [21]	Proneural Like		Glioma Like	
	**Photon**	**Carbon ions**	**Photon**	**Carbon ions**
**Cell growth**	early cytotoxic effect	early cytotoxic effect	cytostatic effect	cytostatic effect
**Cell cycle**	No effect	G0/G1 accumulation	G2/M block	G2/M block
	**Cell metabolite signals**			
**Lipid**	Increase	Increase	No effect	No effect
**Glutamic acid**	No effect	Decrease	No effect	No effect
**GABA**	No effect	Increase	N.D.	N.D.
**Glucose**	Decrease	Decrease	No effect	No effect
**Phosphocreatine**	No effect	Increase	No effect	No effect

## Supplementary Materials

Supplementary Materials can be found at https://www.mdpi.com/1422-0067/21/14/5167/s1. Figure S1: Percentage of line #1 (A) and line #83 (C) photon beam irradiated cells in different phases as obtained by the Annexin V apoptosis assay (Q1: prenecrotic cells, Q2: late apoptotic + necrotic cells; Q3: living cells; Q4: early apoptotic cells). Data from both irradiated and control samples are compared as a function of time after irradiation. Percentage of living #1 (B) and #83 (D) cells, calcuated as Q3/(Q1 + Q2 + Q3 + Q4) * 100. Data from both irradiated and control samples are compared as a function of time after irradiation. Figure S2: Metabolic characteristics of cell line #1 and #83 and their clones Panel A: Box and whisker plots describing the distribution of intensity values of metabolic signals calculated in spectra from line #1 (red boxes) and line #83 cells (green boxes). Reported values are from mobile lipids (ML, A, B- see main text for description), total Creatine (tCr), and Glutathione (GSH). Bar graphs in panels B and C show fold changes between line #1 and line #83 (calculated as log2(FC)). The above mentioned metabolites were evaluated together with glutammine (Gln) and glutammate (Glu). All variations are statistically significant. For Mobile lipids signals a more detailed analysis (panel C) was performed on cross peaks from 2D spectra (A, B, E, F) given their higher statistical significance (*p* < 0.01). Panel D: Box and whiskers plots describing the distribution of intensity values of metabolic signals calculated in spectra from line #83 (green boxes) and its clones (yellow boxes). Reported values are from mobile lipids (ML, A). Bar graph in panel E shows fold changes between line #83 and its clones. Mobile lipid cross peaks from 2D spectra (A, B, E, F) were evaluated. All variations are statistically significant (*p* < 0.05). Clone analysis for line #1 vs line #1 is not shown because no (statistically) significant differences were found. Figure S3: Metabolic changes observed by mr spectroscopy in line #1 cell spectra. Intensity of metabolic signals from 1D and 2D spectra as a function of time after photon (A, C) and carbon ion (B, D) irradiation. Both irradiated and control values are reported. A, B: mobile lipid signals (ML, from 1D spectra and A peak from 2D spectra); C: Glucose signal; D: Glutammic acid, GABA, Phosphocreatine and Glucose signals.

## Abbreviations

COSY	COrrelation SpectroscopY
CSC	Cancer Stem Cells
DSB	Double-Strand Breaks
FA	Fatty Acid
GABA	Gamma-Aminobutyric Acid
GBM	Glioblastoma multiforme
glc	glucose
gln	glutamine
glu	glutamic acid
GSC	Glioblastoma Stem-like Cells
GSf-like	Glioblastoma full stem-like phenotype,
GSH	Glutathione
GSr-like	Glioblastoma restricted stem-like phenotype
IDHs	Isocitrate DeHydrogenases
Lac	Lactate
LET	Linear Energy Transfer
ML	Mobile Lipids
MRS	Magnetic Resonance Spectroscopy
mTOR	Mammalian Target Of Rapamycin
PBS	Phosphate-buffered saline
PCA	Principal Component Analysis
pCr	Phosphocreatine
RBE	Relative Biological Effectiveness
RT	Radiotherapy
tCr	total Creatine
