# Supplementary material for: Different Mechanisms Underlie the Metabolic Response of GBM Stem-Like Cells to Ionizing Radiation: Biological and MRS Studies on Effects of Photons and Carbon Ions
Source: Int J Mol Sci. 2020 Jul 21;21(14):5167. doi: 10.3390/ijms21145167 (PMC7404344; doi:10.3390/ijms21145167)
Supplement: Supplementary file 1 [file ijms-21-05167-s001.zip › FigureS1.pdf]

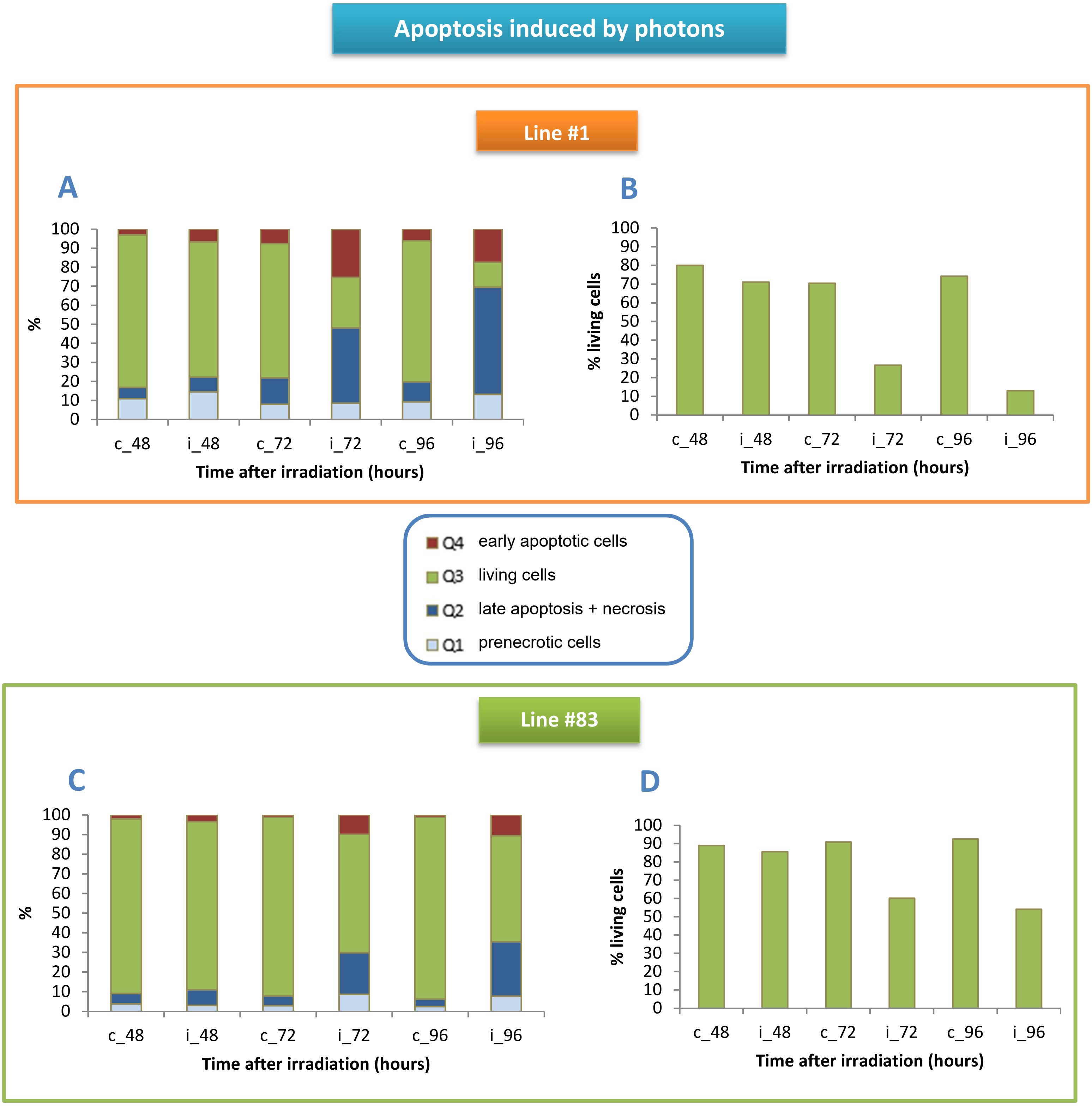

Figure S1.

Percentage of line #1 (A) and line #83 (C) photon beam irradiated cells in different phases as obtained by the Annexin V apoptosis assay (Q1 : prenecrotic cells, Q2: late apoptotic + necrotic cells; Q3: living cells; Q4: early apoptotic cells). Data from both irradiated and control samples are compared as a function of time after irradiation.

Percentage of living #1 (B) and #83 (D) cells, calcuated as Q3/(Q1+Q2+Q3+Q4)*100. Data from both irradiated and control samples are compared as a function of time after irradiation.
