# Supplementary material for: Different Mechanisms Underlie the Metabolic Response of GBM Stem-Like Cells to Ionizing Radiation: Biological and MRS Studies on Effects of Photons and Carbon Ions
Source: Int J Mol Sci. 2020 Jul 21;21(14):5167. doi: 10.3390/ijms21145167 (PMC7404344; doi:10.3390/ijms21145167)
Supplement: Supplementary file 1 [file ijms-21-05167-s001.zip › FigureS2.pdf]

Metabolic characteristics of cell line #1 and #83 and their clones

Panel A: Box and whisker plots describing the distribution of intensity values of metabolic signals calculated in spectra from line #1 (red boxes) and line #83 cells (green boxes). Reported values are from mobile lipids (ML, A, B- see main text for description), total Creatine (tCr), and Glutathione (GSH).

Bar graphs in **panels B and C** show fold changes between line #1 and line #83 (calculated as log2(FC)). The above mentioned metabolites were evaluated together with glutammine (Gln) and glutammate (Glu).

All variations are statistically significant. For Mobile lipids signals a more detailed analysis (panel C) was performed on cross peaks from 2D spectra (A, B, E, F) given their higher statistical significance ($p<0.01$).

Panel D: Box and whiskers plots describing the distribution of intensity values of metabolic signals calculated in spectra from line #83 (green boxes) and its clones (yellow boxes). Reported values are from mobile lipids (ML, A).

Bar graph in **panel E** shows fold changes between line #83 and its clones. Mobile lipid cross peaks from 2D spectra (A, B, E, F) were evaluated. All variations are statistically significant ($p<0.05$).

Clone analysis for line #1 vs line #1 is not shown because no (statistically) significant differences were found.

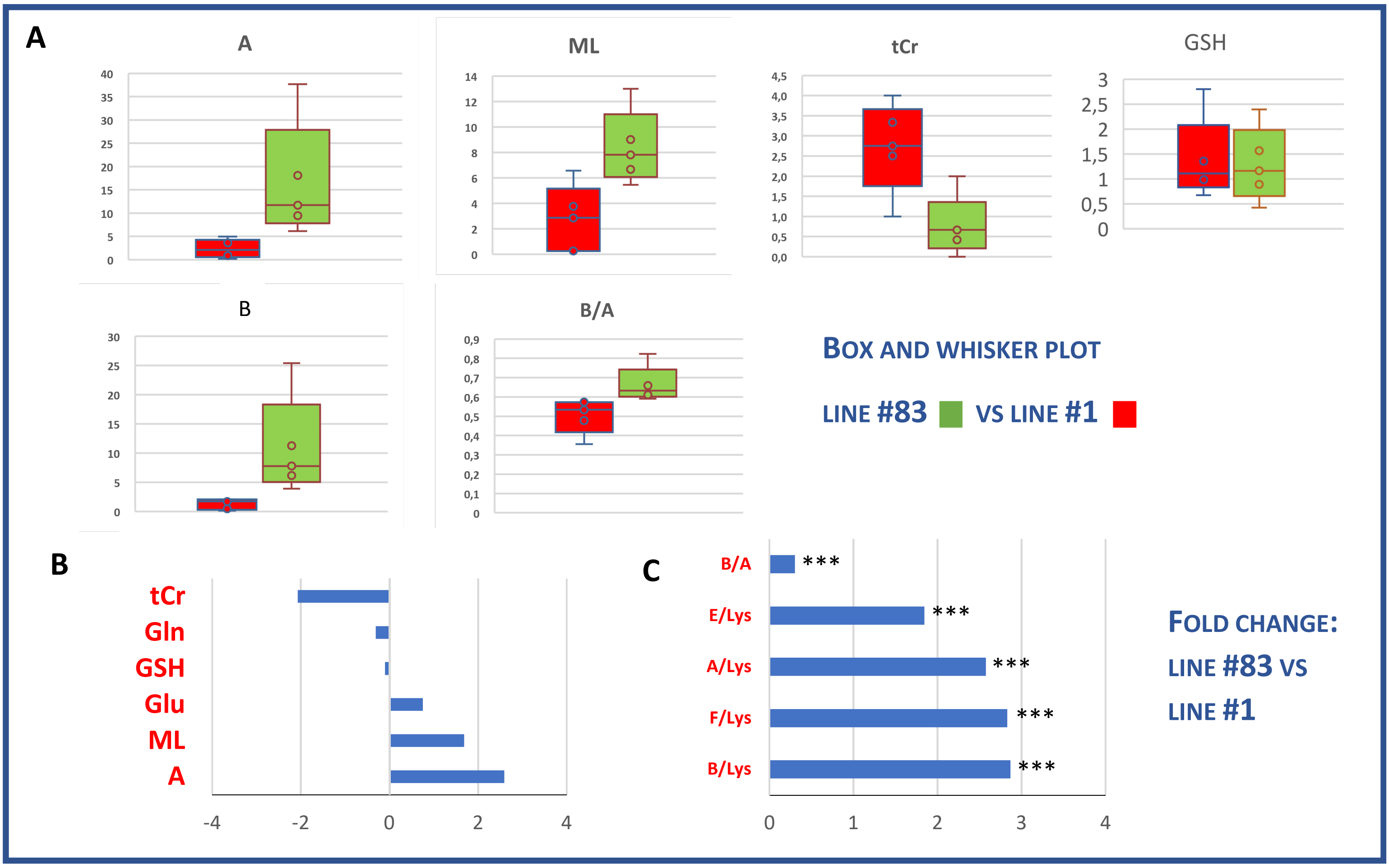
A
A
ML
tCr
GSH
B
B/A
BOX AND WHISKER PLOT
LINE #83 VS LINE #1
B
tCr
Gln
GSH
Glu
ML
A
-4 -2 0 2 4
C
B/A ***
E/Lys ***
A/Lys ***
F/Lys ***
B/Lys ***
0 1 2 3 4
FOLD CHANGE:
LINE #83 VS
LINE #1

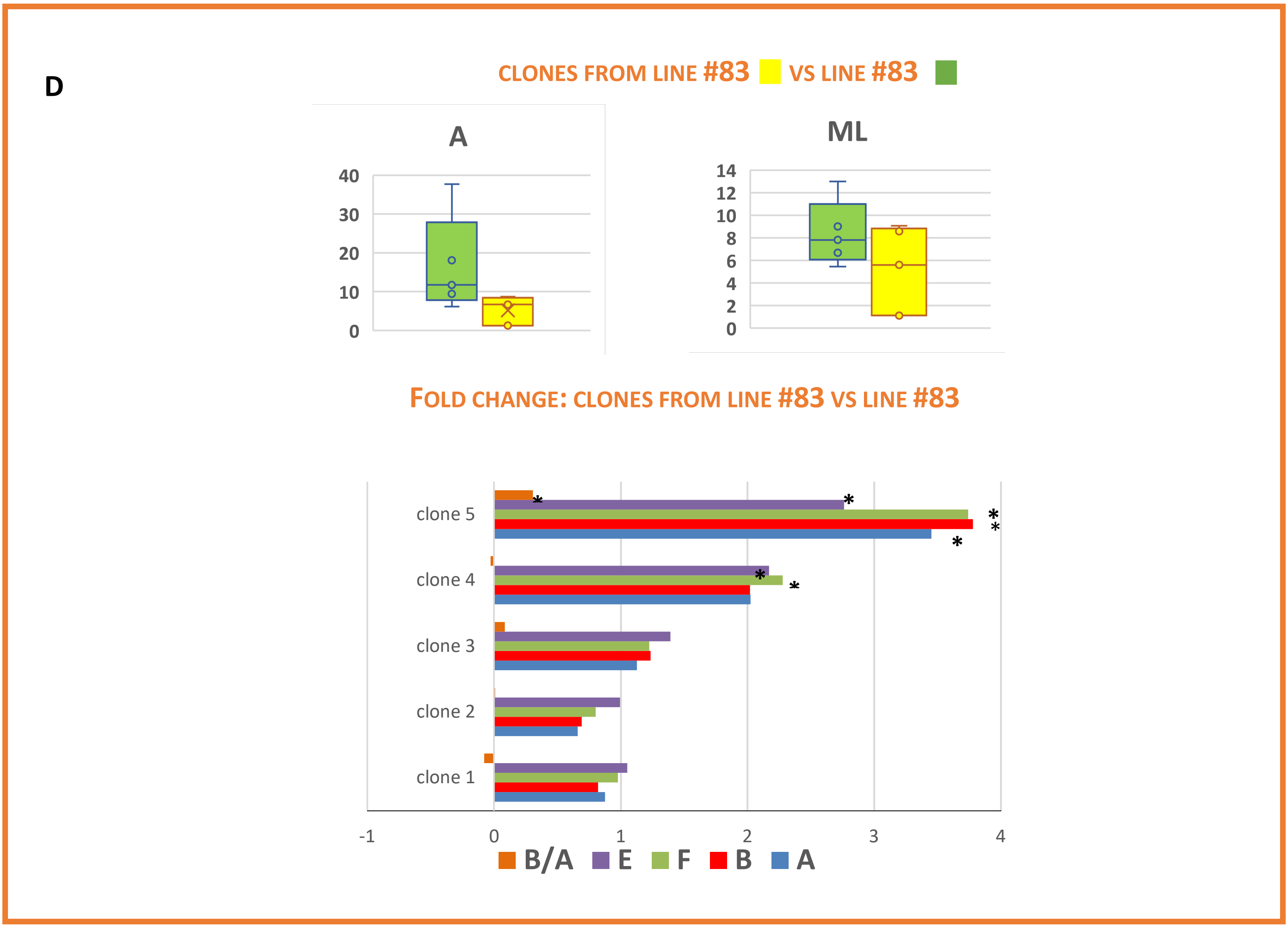
D
CLONES FROM LINE #83 VS LINE #83
A
ML
FOLD CHANGE: CLONES FROM LINE #83 VS LINE #83
clone 5
clone 4
clone 3
clone 2
clone 1
-1 0 1 2 3 4
B/A E F B A
