# Supplementary material for: Different Mechanisms Underlie the Metabolic Response of GBM Stem-Like Cells to Ionizing Radiation: Biological and MRS Studies on Effects of Photons and Carbon Ions
Source: Int J Mol Sci. 2020 Jul 21;21(14):5167. doi: 10.3390/ijms21145167 (PMC7404344; doi:10.3390/ijms21145167)
Supplement: Supplementary file 1 [file ijms-21-05167-s001.zip › FigureS3.pdf]

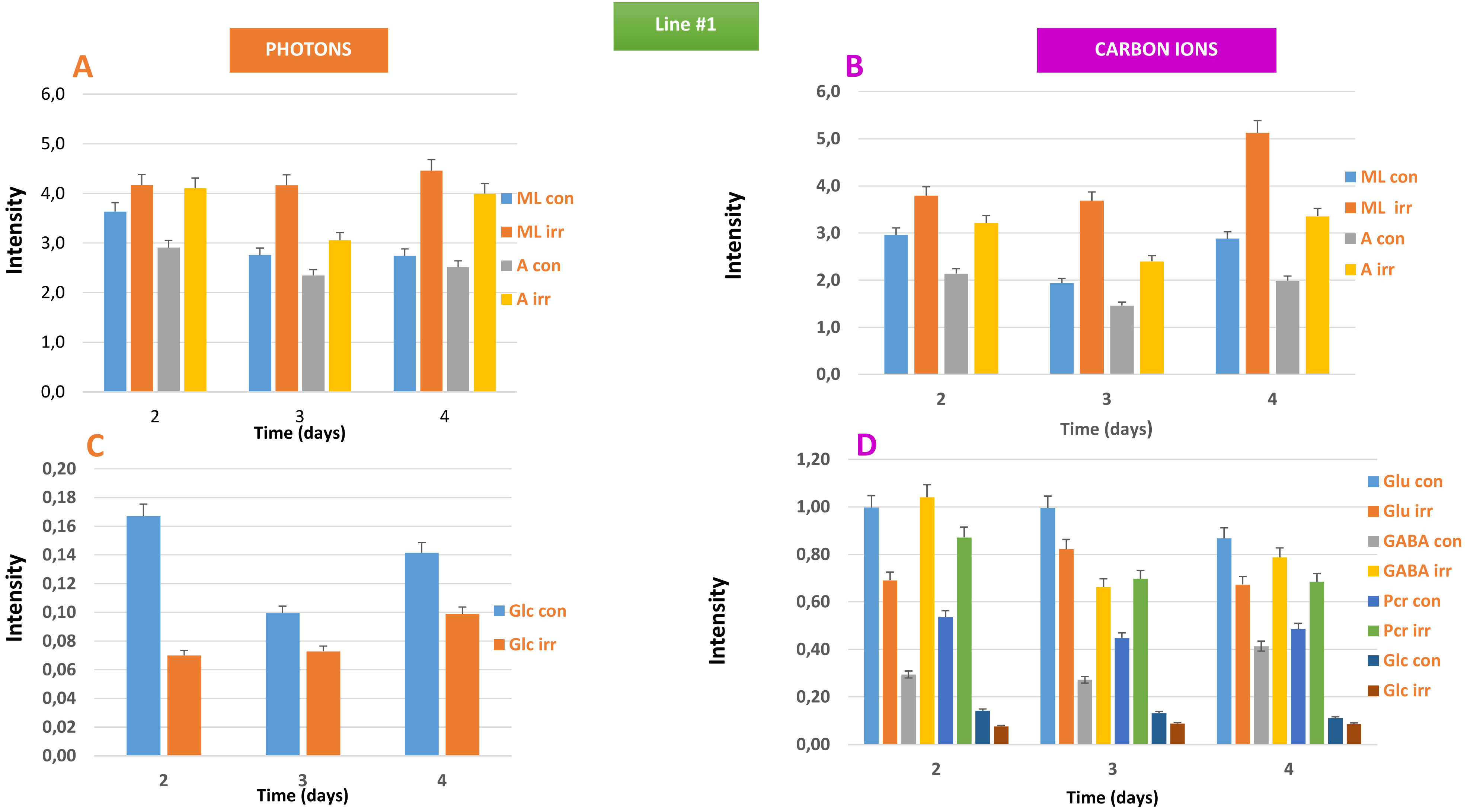

Metabolic changes observed by mr spectroscopy in line #1 cell spectra.
Intensity of metabolic signals from 1D and 2D spectra as a function of time after photon (A, C) and carbon ion (B, D) irradiation. Both irradiated and control values are reported. A, B: mobile lipid signals (ML, from 1D spectra and A peak from 2D spectra); C: Glucose signal; D: Glutammic acid, GABA, Phosphocreatine and Glucose signals.
